# Rheological and Physicochemical Properties of Mayonnaise Enriched with Functional Vegetable Oils: A Comparative Screening Study

**DOI:** 10.3390/foods15122184

**Published:** 2026-06-17

**Authors:** Shakhnozakhon Gaipova, Umrbek Mavlanov, Tomasz Pawel Czaja

**Affiliations:** 1 Department of Food and Perfumery-Cosmetic Products, Tashkent Institute of Chemical Technology, Navoi 32, Tashkent 100011, Uzbekistan; charosgaipova@gmail.com; 2 Department of Food Science, University of Copenhagen, Rolighedsvej 26, 1958 Frederiksberg C, Denmark; umrbek@food.ku.dk

**Keywords:** mayonnaise, oil enrichment, functional oil, fats, edible oils

## Abstract

Sixteen functional vegetable oils were incorporated at a 20% substitution level into a standard mayonnaise formulation to assess the impact of fatty acid composition on physicochemical, textural, rheological, and microstructural properties. Color analysis revealed substantial variation in yellowness (b* = 11.37–30.08) and lightness (L* = 74.39–82.31), while pH remained unaffected across all formulations (3.3–3.6). Texture analysis demonstrated that PUFA-rich oils, particularly linseed, thistle, and corn, produced markedly lower consistency values (17.02–18.68 N·s) compared to MUFA-rich counterparts (up to 69.95 N·s), indicating weaker interfacial network organization. All formulations exhibited non-Newtonian shear-thinning behavior described by the power law model (K = 90.12–130.63 Pa·s^n^; n = 0.162–0.249). Low-field NMR relaxometry identified three distinct proton populations reflecting differences in proton mobility, while diffusometry revealed mean droplet radii ranging from 2.653 µm (pomegranate oil) to 3.203 µm (linseed oil). Pearson correlation and principal component analysis (PCA) confirmed fatty acid unsaturation as the primary driver of droplet size distribution and textural differentiation among formulations. The study was designed as an exploratory screening of single-batch formulations, and the results are presented descriptively to identify comparative trends among the different oils. Linseed, walnut, and pomegranate oils showed favorable compositional profiles for mayonnaise reformulation, combining favorable PUFA-to-SFA ratios with acceptable emulsion stability and rheological performance.

## 1. Introduction

Mayonnaise is a classic oil-in-water (O/W) emulsion comprising 70–80% vegetable oil emulsified primarily by phospholipids from the egg yolk. They stabilized fine oil droplets (1–10 um) responsible for mayonnaise characteristic creamy structure, viscoelastic properties and broad consumer acceptance. Traditional production involves the gradual incorporation of oil into an aqueous phase containing egg yolk as an emulsifying agent and acidulants such as vinegar or lemon juice. This process results in a semi-solid emulsion with a pH in the range of 3.5–4.5, which resists creaming and coalescence due to effective control of interfacial tension at the oil–water interface [1,2,3].

In Uzbekistan, mayonnaise is widely used in salads and festive dishes, per capita consumption data remain unavailable. However, rapidly growing domestic production reaching 1650 tons (increase + 7% y/y) with total annual capacity of approx. 5800 tons paralleled by substantial imports of 18,590 tons primarily from Russia, Kazakhstan and Turkey [4]. Taken together, these figures suggest that mayonnaise is a relevant and expanding product category in the local market, corresponding to an approximate availability of more than 0.7 kg per person per year based on total annual domestic production plus imports divided by the national population. Classic mayonnaise formulations rely on their high fat content to achieve desirable viscosity and mouthfeel; however, alongside favorable sensory attributes, their nutritional implications raise important health-related concerns. High dietary intake of saturated fats and cholesterol has been associated with disturbances in lipid metabolism, including elevated total and low-density lipoprotein (LDL) cholesterol levels, which are well-established risk factors for cardiovascular diseases, as well as obesity and diabetes [5,6]. This issue is particularly relevant in Uzbekistan, where cardiovascular diseases remain among the leading causes of mortality.

Consequently, increasing awareness of diet-related health risks has driven food technologists and nutritionists to reformulate mayonnaise products toward improved nutritional quality. Current strategies focus on modifying the fatty acid profile through the incorporation of oils rich in unsaturated and omega-3 fatty acids, reducing total fat content via partial fat replacement, enriching products with functional ingredients such as antioxidants, phytosterols, and dietary fiber, and lowering cholesterol levels through reduced-egg or egg-free formulations [7,8]. These approaches aim to maintain the desirable sensory properties of mayonnaise while addressing public health concerns associated with excessive fat consumption.

Several studies have investigated the incorporation of functional oils into mayonnaise formulations to improve their nutritional value. Replacement of conventional vegetable oil with flaxseed oil has been shown to significantly increase omega-3 fatty acid content while maintaining acceptable sensory quality and emulsion stability during storage [9]. Similarly, blends of chia and fish oil microencapsulated via spray-drying have been successfully incorporated into mayonnaise, achieving enhanced omega-3 enrichment alongside improved oxidative stability [10]. Extra-virgin olive oil has been evaluated as a partial oil substitute, yielding mayonnaise with modified physical and structural properties attributable to differences in fatty acid composition and minor bioactive compounds [11]. Beyond oil replacement, enrichment with plant-based antioxidants has been shown to enhance phenolic bio-accessibility and oxidative stability of the final product [12]. Furthermore, walnut oil-enriched mayonnaise characterized by a high n-3/n-6 PUFA ratio demonstrated acceptable rheological and structural properties [13], while black cumin oil incorporation significantly improved oxidative stability through its thymoquinone content [14]. Shrimp oil, introduced using fish myofibrillar protein as an egg yolk substitute, has also been evaluated for its effects on physical, rheological, and sensory properties [15]. Collectively, these studies confirm that partial replacement of conventional oils with functional alternatives is a viable strategy for nutritional enrichment, though oxidative stability and emulsion integrity remain primary technological challenges [16].

The sixteen oils were selected to represent a broad spectrum of fatty acid compositions, ranging from high-PUFA oils such as linseed, walnut, pomegranate, grapeseed, sunflower, and hemp, to oils with intermediate and high-MUFA contents, such as sesame, olive, avocado, almond, and peanut. In this study, the term “functional oils” refers to vegetable oils that, beyond their energy contribution, provide biologically relevant lipid fractions, particularly polyunsaturated fatty acids, with potential nutritional value. The selection of oils allowed the effects of different lipid profiles to be compared under identical mayonnaise formulation conditions. This study investigates the potential for developing healthier mayonnaise formulations by replacing 20% of conventional vegetable oil with functional oils rich in PUFAs, MUFAs, and omega-3 fatty acids. The reformulated products were evaluated in terms of color, texture, rheological properties, oil droplet size distribution, and pH. These analyses were performed to determine whether nutritional enrichment could be achieved without substantially altering the key physicochemical characteristics of conventional mayonnaise.

The findings aim to support lipid reformulation strategies that reduce saturated fat content while preserving product quality. Unlike previous studies that focused on single oil substitutions, or limited number of formulation variants, the present work was designed as an exploratory screening study to compare sixteen oils with distinct fatty acid profiles under identical mayonnaise formulation conditions. This approach combines conventional physicochemical analysis with low-field and benchtop NMR-based measurements to identify broad compositional trends and promising candidate oils for future validation studies.

## 2. Materials and Methods

### 2.1. Materials

All ingredients used for the preparation of the mayonnaise formulations were purchased from local suppliers in Copenhagen, Denmark, and Tashkent, Uzbekistan. Various vegetable oils, including rapeseed, sunflower, grapeseed, pomegranate, almond, cumin, peanut, soybean, olive, pumpkin, walnut, avocado, hemp, sesame, lineseed, thistle (safflower), and corn oils, were purchased from local markets in Copenhagen, Denmark. Additional ingredients, including sugar, salt, vinegar (6%), and mustard, were obtained from local retail outlets in Copenhagen, Denmark. Egg yolk powder and citric acid were purchased from Foods Impex Group LLC (Tashkent, Uzbekistan). The oils were commercially available products purchased at the same time from local suppliers and stored in their original sealed containers under refrigeration at 5 °C in the dark immediately after purchase until use. Information available from the product labels, including processing type and organic status, is summarized in Table S1. Peroxide value, acid value, and other oxidative status indicators were not determined in the present study.

### 2.2. Mayonnaise Preparation

**Figure 1:**
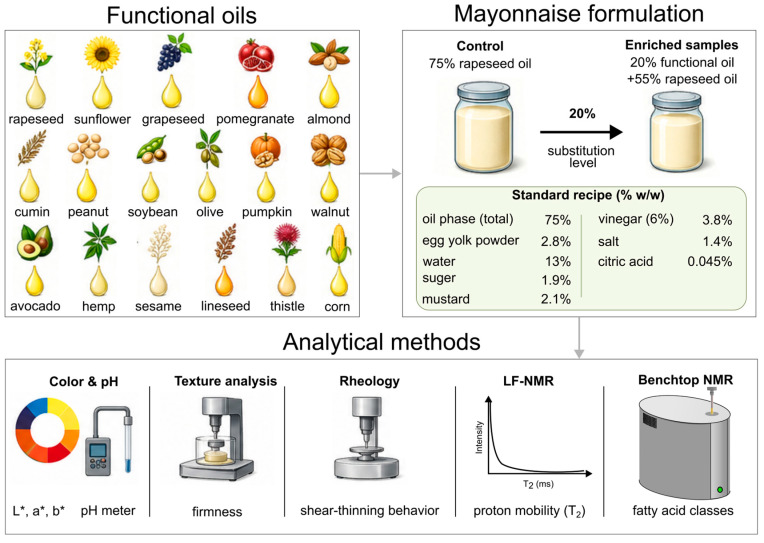
Study design.

Standard mayonnaise samples were prepared in 1000 g batches using a standardized formulation (% *w*/*w*): 75% rapeseed oil, 2.8% egg yolk powder, 13% distilled water, 1.9% sugar, 2.055% mustard, 0.045% citric acid, 1.4% salt, and 3.8% vinegar (6% acidity) [17]. Enriched samples were formulated by replacing 20% of the rapeseed oil with functional oils (20% functional oil and 55% rapeseed oil) (Figure 1). The 20% substitution level was selected as a practical partial replacement level, allowing the effects of the functional oils to be evaluated while keeping the mayonnaise within a conventional formulation range. This level was also considered appropriate because some of the oils investigated are relatively expensive and have distinctive sensory characteristics, such as intense flavor or aroma, making full replacement less feasible for routine product development. Samples were processed using a Stephan Universal Machine UMC5 lab-scale mixer in a two-stage procedure under standardized conditions. In Stage 1, egg yolk powder, salt, sugar, citric acid, and mustard were pre-mixed without vacuum at 1500 rpm for 2 min, followed by vacuum application −0.9 bar with mixing time 1 min. In Stage 2, rapeseed oil and for enriched samples, the corresponding functional oil were gradually added under vacuum, followed by the addition of vinegar at 3000–4500 rpm over a total processing time of 4 min. Prepared mayonnaises were packaged in sterile containers and stored in the dark at 4 °C for 24 h prior to analysis. Each mayonnaise formulation was prepared as a single batch, as the present study was designed as an exploratory screening experiment rather than a replicated batch study. For each analytical method, one measurement was performed per sample; therefore, technical replicates were not included. Before texture and rheological analyses, samples were equilibrated to 25 °C, analyzed in randomized order, and visually checked for emulsion stability.

### 2.3. Colour Measurements

Color measurements were conducted using a Konica Minolta CR-400 spectrophotometer (Osaka, Japan), calibrated in the CIE L*a*b* color space (D65 illuminant, 10° observer, SCE mode). Here, L* denotes lightness (0 = black to 100 = white), a* the red-green axis (−green, +red), and b* the yellow-blue axis (−blue, +yellow). Three measurements were taken per sample and the average was reported. The instrument was calibrated using the standard white/black calibration procedure before each measurement session. Samples were analyzed after reaching room temperature following storage at 5 °C. ΔE values were calculated relative to the control mayonnaise.

### 2.4. pH

The pH was determined by dispersing 10 g of sample in 90 mL distilled water, followed by homogenization until emulsion stability was achieved. The suspension was then measured using a calibrated Mettler-Toledo pH/ion bench meter (model SC S220-B, Columbus, OH, USA) with a glass electrode immersed directly into the sample.

### 2.5. Texture Analysis

The textural properties of the mayonnaise samples were determined using a back-extrusion test performed with a Texture Analyzer TA. XTplus (Stable Micro Systems, Godalming, UK). The instrument was equipped with a 35 mm-diameter cylindrical disc and a load cell of 5 kg-f. The samples were placed in cylindrical containers (inner diameter: 50 mm; height: 75 mm) and analyzed at 25 °C. Three measurements were performed for each sample, and the mean value was reported. Before testing, the samples were visually inspected to confirm the absence of air bubbles. All samples were filled to the same height using the same container. The test consisted of a compression stroke to 75% sample deformation. The pre-test, test, and post-test speeds were set to 1.0, 1.0, and 10.0 mm/s, respectively. The samples were analyzed according to the mayonnaise measurement protocol available in the instrument software. From the recorded force–time curves, the following parameters were calculated using Exponent software (version 8.0.16, Stable Micro Systems, UK): firmness (maximum force recorded during the compression phase, N), consistency (area under the positive force–time curve during compression, N·s), cohesiveness (area under the negative force–time curve during the probe withdrawal phase, N·s), and work of cohesion (area under the negative force–distance curve during the probe withdrawal phase, N·mm). The method was from Zhao et al. with minor modifications [5].

### 2.6. Rheological Properties

Rheological measurements of mayonnaise samples were performed using a Kinexus Pro rheometer (model KNX 2100; Malvern Instruments, Malvern, UK) equipped with a 50 mm parallel plate geometry and a measurement gap of 1.0 mm, controlled by rSpace software (version 1.75). Steady shear tests were conducted over a shear rate range of 0.1–100 s^−1^, after a pre-shear step of 20 s^−1^ applied for 10 s to ensure structural uniformity. The viscosity data were fitted to the power-law model to obtain the consistency coefficient (K) and flow behavior index (n). The yield stress was defined as the critical stress at which the sample began to deform irreversibly. All rheological measurements were carried out at 25 °C.

### 2.7. Proton Mobility and Droplet Size Distribution (DSD)

Proton mobilitiy in prepared mayonnaises was determined using low-field nuclear magnetic resonance (LF-NMR) with an MQR Spectro-P spectrometer (Oxford Instruments, Oxfordshire, UK) operating at 20 MHz (0.47 T). The instrument was controlled by the Oxford Instruments Application Development software (version 0.10.8). Samples were cut into uniform portions and placed in 18 mm glass NMR tubes prior to measurement. Spin–spin relaxation ($T_{2}$) measurements were acquired at 5 °C using the Carr–Purcell–Meiboom–Gill (CPMG) pulse sequence. The experimental parameters were as follows: inter-echo time (τ) = 300 µs, recycle delay = 8 s, 16 scans, and 10,000 echoes with a receiver gain of 5 dB. The transverse relaxation time components ($T_{2n}$) and their relative signal amplitudes were obtained by multiexponential fitting of the decay curve I(t) using an in-house MATLAB script (R2024a, MathWorks, Natick, MA, USA), according to the model:

(1) $$I\left(t\right)=\sum_{n=1}^{N}M_{n}*e^{-t/T_{2n}}\;$$

where I(t) is the measured signal intensity, N is the number of relaxation components, and $M_{n}$ corresponds to the relative proton population associated with the relaxation time $T_{2n}$. The number of components was determined based on the analysis of fitting residuals. Droplet size distribution (DSD) was determined using LF-NMR, an established and widely used technique for comparative characterization of droplets in emulsion systems, based on restricted self-diffusion analysis. A $T_{1}$ relaxation filter was applied to suppress signals from the continuous phase, followed by a pulsed-field gradient (PFG) diffusometry sequence used to characterize the dispersed phase. Diffusion attenuation data were fitted using the Balinov restricted diffusion model assuming a lognormal droplet size distribution, from which the mean droplet radius and volumetric size fractions (<2, 2–5, and 5–10 µm) were derived [18]. All NMR experiments were carried out at 5 °C.

### 2.8. Benchtop ^1^H NMR Acquisition and Processing

For benchtop ^1^H NMR analysis, 250 µL of pure oil was mixed with 400 µL deuterated chloroform (CDCl_3_) containing 0.03% tetramethylsilane (TMS) as an internal reference. The mixture was transferred into 5 mm NMR tubes (Wilmad-LabGlass, Vineland, NJ, USA). Spectra were acquired using an 80 MHz benchtop NMR spectrometer (Spinsolve 80 Ultra, Magritek, Aachen, Germany). The spectrometer was initially shimmed using a standard D_2_O/H_2_O reference sample, followed by individual sample shimming prior to each measurement. One-dimensional ^1^H NMR spectra were acquired using the 1Pulse (single-pulse) sequence with ^13^C decoupling enabled. For each sample, 64 scans were collected with an acquisition time of 3.2 s, a repetition delay of 10 s, and a 90° pulse angle. Spectra were recorded over the chemical shift range from −1 to 8 ppm. Spectral processing was conducted in Python (version 3.12).

### 2.9. Quantification of Saturated and Unsaturated Fatty Acids

The quantification of SFA, MUFA, and PUFA was performed using a ^1^H NMR signal integration approach based on the proportional relationship between resonance area and the number of contributing protons. This method has been previously validated for edible oil analysis in our earlier work [19].

The PUFA content was calculated according to:

(2) $$PUFA\;\left(\%\right)=\frac{G}{F}\;\times \;100$$

where F represents the integral of the signal at 2.28–2.36 ppm corresponding to methylene protons adjacent to the carbonyl group of all fatty acids (–C**H_2_**–COO–), used as an internal reference, and G represents the integral of the signal at 2.60–2.95 ppm corresponding to bis-allylic protons (=CH–C**H_2_**–CH=) specific to PUFA.

The total unsaturated fatty acid (UFA) content was determined as:

(3) $$UFA\;\left(\%\right)=\frac{E}{2F}\;\times \;100$$

where E corresponds to the integral of the signal at 1.85–2.23 ppm attributed to allylic protons (–C**H_2_**–CH=CH–) present in all unsaturated fatty acids. The factor of 2 accounts for normalization relative to the reference signal.

The MUFA content was calculated by subtracting the PUFA contribution from total UFA:

(4) $$MUFA\;\left(\%\right)=UFA-PUFA\;$$

Finally, the SFA content was determined as the remaining fraction:

(5) $$SFA\;\left(\%\right)=100-UFA$$

### 2.10. Data Analysis

Multiexponential fitting of LF-NMR T_2_ relaxation decay curves was performed using an in-house script developed in MATLAB (R2024a, MathWorks, USA), as described in Section 2.7. Quantification of fatty acid fractions (SFA, MUFA, PUFA) from ^1^H NMR spectra was conducted using signal integration routines implemented in Python (v3.13). Pearson correlation analysis and principal component analysis (PCA) were performed in Python (v3.12).

## 3. Results and Discussion

### 3.1. Influence of Functional Oil Type and Pigment Composition on Color Attributes and pH of Mayonnaise Emulsions

The color parameters of mayonnaise formulations were determined in the CIE LAB color space (L*, a*, b*) using a D65 illuminant. The control mayonnaise showed L*, a*, and b* values of 81.43, −0.40, and 13.01, respectively. Incorporation of functional oils led to pronounced variations in color attributes, likely influenced by the pigment composition and optical characteristics of the oil phase, as reported in the literature. The lightness (L*) values of experimental samples ranged from 74.4 to 82.3. Mayonnaise formulated with pumpkin, avocado, hemp, thistle, and corn oils exhibited notably lower lightness compared to the control (ΔL* < −4.8; L* < 77.4), indicating a darker appearance attributable to enhanced light absorption by oil-soluble pigments, predominantly chlorophylls and carotenoids characteristic of these oils [20]. Other formulations showed L* values comparable to those of the control sample. Yellow chroma (b*) values varied substantially among samples, ranging from 11.37 to 30.08. Increased yellowness relative to the control (Δb* > +1.5; b* > 14.3) was observed in samples prepared with pomegranate, cumin, olive, pumpkin, avocado, and hemp oils. The highest b* value was recorded for the avocado oil formulation (b* = 30.08; Δb* = +17.07), attributable to the abundance of pheophytins a and b alongside lutein—the dominant carotenoid in avocado oil, accounting for ~70% of its total carotenoid fraction—which impart intense yellow-green hues to food emulsions [21]. The lowest yellowness was observed in the almond oil sample (b* = 11.37), consistent with the low pigment content of refined almond oil. All formulations exhibited negative a* values, indicating a slight greenish hue, with a* values ranging from −3.59 to −0.25. The strongest green shift compared to the control was observed in mayonnaise containing pumpkin oil (Δa* = −3.19), reflecting the exceptionally high chlorophyll content of pumpkin seed oil—which exceeds its carotenoid content sixfold—with lutein and zeaxanthin as the predominant carotenoid species [22]. Detailed colorimetric data are provided in Table S1.

The pH of all mayonnaise formulations remained within a narrow range of 3.3–3.6, indicating that the substitution of rapeseed oil with functional oils had no significant effect on the acidity of the continuous phase, which is primarily determined by the vinegar concentration in the recipe. This demonstrates that pH is largely independent of oil composition and governed by the aqueous phase of the emulsion. The slightly lower pH observed in the sesame oil formulation (3.3) may reflect minor interactions between sesame oil constituents, including lignans such as sesamin and sesamolin and the aqueous phase. Overall, these results highlight that functional oil incorporation significantly modulates the visual attributes of mayonnaise through pigment-driven optical effects, while preserving the fundamental acid stability of the emulsion system.

### 3.2. Fatty Acid Profile of Vegetable Oils

The fatty acid profiles of the analyzed oils varied considerably across samples (Table 1). This variability reflects fundamental differences in lipid composition [23], which are expected to influence both the nutritional value and functional behavior of the resulting emulsions. Oils with the highest PUFA content included walnut (75%), pomegranate (75%), grapes (74%), linseed (73%), and sunflower (69%), indicating their relatively high levels of polyunsaturated fatty acids. These oils are characterized by a high degree of unsaturation, which is associated with improved nutritional profiles but may also influence oxidative stability [24] and emulsion structure. In contrast peanut (17%), olive (11%) and avocado (11%) exhibited the lowest PUFA fractions. Such oils are comparatively less enriched in essential fatty acids, and their fatty acids profiles are therefore less dominated by polyunsaturated components. Regarding MUFA content, olive (73%), avocado (73%), and almond (66%) were the richest sources, consistent with their well-established high oleic acid content. High-MUFA oils are typically associated with enhanced oxidative stability and distinct interfacial properties, which may affect droplet formation and rheological behavior in emulsified systems.

Walnut (8%) and pomegranate (13%) showed the lowest MUFA levels. In terms of SFA content, pumpkin (22%), peanut and cumin (19%) had the highest saturated fat fractions among the analyzed oils, while linseed (9%) and rapeseed (8%) contained the least saturated fatty acids. Higher SFA levels are generally associated with increased lipid rigidity [25], which may contribute to differences in texture and flow behavior of the final mayonnaise formulations. From a nutritional reformulation perspective, linseed, walnut, and pomegranate oils stood out as favorable candidates due to their fatty combination of high PUFA and low SFA content, which aligns with the goal of improving the fatty acid profile of mayonnaise while reducing saturated fat intake (Table 1). However, PUFA-rich oils may also be more susceptible to oxidation, which should be considered when interpreting their suitability for mayonnaise reformulation. Overall, the wide compositional diversity among the selected oils provides a robust framework for systematically investigating how fatty acid composition governs the physicochemical and microstructural properties of mayonnaise emulsions.

### 3.3. Influence of Functional Oil Composition on Textural Properties of Mayonnaise Emulsions

The textural properties of mayonnaise samples formulated with different vegetable oils were evaluated using a texture analyzer, and the results are presented in Table S2. Marked differences in all textural parameters were observed among formulations, indicating that oil composition plays a critical role in determining the mechanical behavior of the emulsion network. Firmness values ranged from 5.89 N (cumin oil) to 9.09 N (sunflower oil), reflecting differences in the structural firmness of the emulsions associated with the varying fatty acid profiles of the oils used. Higher firmness values suggest a more rigid and interconnected droplet network, whereas lower values indicate a weaker structural organization within the emulsion. Consistency values showed the most pronounced variation among all textural parameters, ranging from 17.02 N·s (linseed oil) to 69.95 N·s (sunflower oil). This parameter was particularly sensitive to oil type, highlighting its relevance as an indicator of interfacial interactions and continuous-phase structuring. Notably, mayonnaise prepared with linseed, thistle, and corn oils exhibited markedly lower consistency values (17.02–18.68 Ns) compared to all other formulations (43.27–69.95 Ns), indicating weaker mechanical resistance of the emulsion network. This trend was associated with the PUFA-rich composition of these oils, although the molecular mechanism was not directly investigated in the present study. Work of cohesion ranged from 5.26 N·mm (pomegranate oil) to 7.11 N·mm (sunflower oil), while cohesiveness values ranged from 4.62 N·s (thistle and corn oils) to 7.07 N·s (grapeseed oil), indicating differences in the resistance of the emulsion network to deformation and the energy required for structural recovery. Higher work of cohesion and cohesiveness values indicate a more elastic and resilient structure, whereas lower values reflect weaker internal bonding within the emulsion matrix. The control mayonnaise exhibited firmness of 6.25 N, consistency of 51.06 N·s, work of cohesion of 5.84 N·mm, and cohesiveness of 5.45 N·s, representing intermediate textural characteristics among all tested formulations. Overall, these findings demonstrate that fatty acid composition, particularly the degree of unsaturation, significantly influences the mechanical properties of mayonnaise by modulating interfacial interactions and network formation within the emulsion system.

### 3.4. Influence of Functional Oil Composition on Rheological Behavior of Mayonnaise Emulsions

The flow behavior of mayonnaise samples formulated with different vegetable oils was described using the Ostwald–de Waele (power law) model. The consistency index (K), flow behavior index (n), and coefficients of determination (R^2^) are presented in Table S3. All formulations, including the control sample, exhibited non-Newtonian, shear-thinning behavior, as evidenced by flow behavior index (n) values ranging from 0.162 to 0.249, indicating pronounced pseudoplastic behavior across all samples. The cconsistency index (K) ranged from 90.12 to 130.63 Pa·s^n^. The highest K value was observed in mayonnaise prepared with grapeseed oil (130.63 Pa·s^n^), whereas the lowest values were recorded for pomegranate (90.12 Pa·s^n^), cumin (92.10 Pa·s^n^), sesame and walnut (92.05 Pa·s^n^) oil formulations. The control mayonnaise exhibited a K value of 118.93 Pa·s^n^, while the remaining samples showed intermediate K values. All rheological measurements were conducted over a shear rate range of 0.124–99.24 s^−1^, with 31 data points collected per sample. The power law model provided an excellent fit to the experimental data, with R^2^η values ranging from 0.984 to 0.997 and R^2^τ values ranging from 0.951 to 0.992, respectively, where R^2^η and R^2^τ denote the coefficients of determination for the viscosity–shear rate and shear stress–shear rate fits, confirming high agreement between experimental and predicted values for all formulations. Detailed rheological parameters are provided in Table S3.

### 3.5. Droplet Size Distribution and Proton Mobility in Mayonnaise Emulsions

LF-NMR T_2_ analysis identified three distinct proton populations in all mayonnaise formulations, reflecting water fractions of differing mobility. This multi-component relaxation behavior provides insight into the microstructural organization of the emulsion, particularly the distribution and confinement of water within different structural domains. The fast-relaxing component (T_21_: 25.47–40.62 ms) is attributed to water tightly associated with the protein–lecithin interfacial layer surrounding oil droplets, the intermediate component (T_22_: 66.46–105.33 ms) corresponds to water in the structured continuous phase, and the slow-relaxing component (T_23_: 169.56–262.73 ms) represents the most mobile, bulk-like water fraction. Shorter T_2_ values indicate increased molecular restriction, reflecting stronger interactions between water molecules and the surrounding matrix. Thistle and corn oil formulations exhibited markedly lower T_2_ values across all three populations compared to the control (T_21_: 38.24 ms; T_23_: 234.96 ms), indicating reduced proton mobility and altered water organization within the emulsion. This suggests the formation of a more tightly packed interfacial layer and enhanced structural integrity of the emulsion. Conversely, soybean, olive, pumpkin, and walnut oil mayonnaises displayed the highest T_23_ values (>256 ms), suggesting greater free water mobility and a less structured matrix. Such behavior indicates weaker water–matrix interactions and a more open microstructure with reduced interfacial confinement. The mean droplet radius (D) ranged from 2.653 µm (pomegranate oil) to 3.203 µm (linseed oil), with all formulations dominated by the 2–5 µm fraction (63.71–76.34%). These results confirm that all systems formed fine emulsions; however, subtle differences in droplet size distribution significantly influenced structural properties. Pomegranate oil yielded the finest emulsion structure, evidenced by the lowest mean radius and minimal proportion of large droplets (5–10 µm: 0.70%), whereas avocado and olive oil formulations showed the highest fractions of large droplets (6.40% and 5.15%, respectively), suggesting lower emulsification efficiency potentially related to the elevated viscosity of these oils. The formation of larger droplets in MUFA-rich oils may be associated with increased oil-phase viscosity and reduced droplet breakup during emulsification, leading to coarser dispersions. Taken together, formulations with smaller droplets and lower T_23_ values—particularly thistle and corn oil variants—indicate a more structured interfacial network, while larger-droplet, high-T_2_ formulations such as olive and avocado may present a less cohesive emulsion matrix.

### 3.6. Multivariate Data Analysis

**Figure 2:**
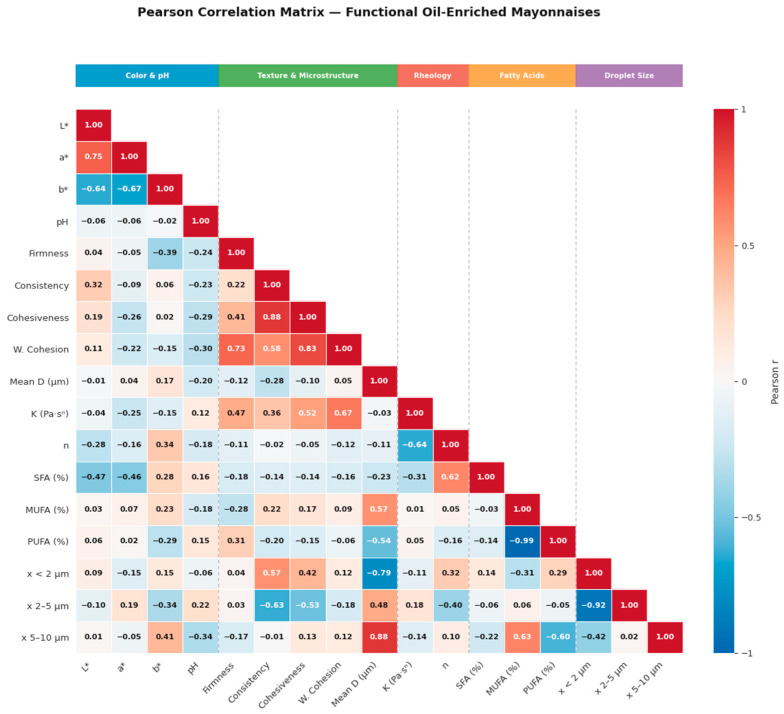
Pearson correlation matrix of physicochemical, textural, rheological, and fatty acid composition parameters of mayonnaises enriched with 20% of different vegetable oils.

Pearson correlation analysis was performed to identify cross-domain relationships between colour, texture, rheology, particle size, and fatty acid composition of enriched mayonnaises (Figure 2). The most relevant inter-group associations involved fatty acid composition and emulsion microstructure: MUFA content showed a positive correlation with the coarse droplet fraction 5–10 µm (r = 0.63), while PUFA exhibited an opposing negative trend (r = −0.60), suggesting that oils rich in monounsaturated fatty acids promoted the formation of larger droplets, potentially due to differences in interfacial tension or emulsifier interactions. Furthermore, mean radius correlated negatively with PUFA (r = −0.54) and positively with MUFA (r = 0.57), reinforcing the notion that the degreee of fatty acid unsaturation influenced droplet size distribution in the enriched emulsions. Regarding rheological behaviour, SFA content correlated positively with the flow behaviour index n (r = 0.62), indicating that mayonnaises enriched with oils higher in saturated fatty acids exhibited less pronounced shear-thinning behaviour. SFA also showed negative correlations with L* (r = −0.47) and a* (r = −0.46), suggesting an influence of fatty acid composition on the optical properties of the emulsion. The b* parameter correlated negatively with pH (r = −0.34), indicating that more acidic formulations tended to exhibit reduced yellowness. These cross-parameter relationships demonstrate that the choice of enriching oil affected not only the nutritional profile but also the microstructure, flow behaviour, and colour of the final mayonnaise product.

**Figure 3:**
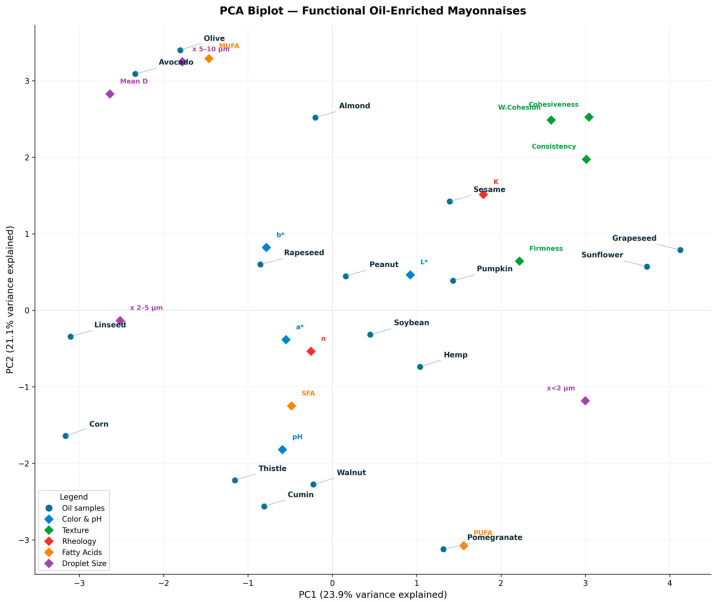
A biplot of physicochemical, textural, rheological, and fatty acid composition parameters of mayonnaises enriched with 20% of different vegetable oils. Circles represent oil samples; diamonds represent physicochemical parameters colored by variable group.

Principal component analysis (PCA) was performed to integrate physicochemical, textural, rheological, emulsion microstructure, and fatty acid composition data into a single multivariate framework (Figure 3). The first two principal components accounted for 23.9% (PC1) and 21.1% (PC2) of total variance, respectively, yielding a cumulative explained variance of 45.0%.

PC1 was primarily associated with fatty acid composition and droplet size parameters. Oils rich in MUFA—notably olive and avocado—were positioned in the upper-left quadrant of the biplot, co-localizing with the Mean D and 5–10 µm loadings, consistent with the positive correlation previously identified between MUFA content and large droplet fraction (r = 0.63). Conversely, PUFA-rich formulations—pomegranate, walnut, and linseed—were displaced towards the right of the ordination space, in proximity to the x < 2 µm loading, in agreement with the negative association between PUFA content and mean droplet radius (r = −0.54). These results indicate that the degree of fatty acid unsaturation was a primary determinant of emulsion droplet size distribution.

PC2 separated formulations primarily based on textural parameters. Firmness, Consistency, Cohesiveness, and Work of Cohesion formed a distinct cluster in the upper-right region of the biplot, with sunflower and grapeseed oil formulations projecting towards this cluster, reflecting their elevated consistency values. Linseed, corn, and thistle oil formulations were displaced towards the lower-left quadrant, consistent with their markedly reduced consistency values (17.02–18.68 N·s) and proton mobility observed by LF-NMR.

Color parameters (L*, a*, b*) and pH were positioned near the center of the ordination space, indicating a moderate contribution to overall sample differentiation. The b* loading was positioned in proximity to pigment-rich formulations (avocado, pumpkin), reflecting the dominant role of carotenoids and chlorophylls in yellowness variation, in agreement with the results reported in Section 3.1. Taken together, the PCA ordination confirmed that functional oil selection simultaneously modulates multiple quality attributes of mayonnaise, with fatty acid composition acting as the primary driver of microstructural and textural differentiation among formulations.

## 4. Conclusions

The incorporation of functional oils into mayonnaise formulations significantly influenced the physicochemical, textural, rheological, and microstructural properties of the final product. Color analysis demonstrated that the type of oil used determined the appearance of the emulsion, with avocado, pumpkin, and hemp oils producing markedly darker and more yellow-chromatic products due to their native carotenoid and chlorophyll content. All formulations exhibited non-Newtonian, shear-thinning behavior, with the consistency index (K) varying between 90.12 Pa·s^n^ (pomegranate oil) and 130.63 Pa·s^n^ (grapeseed oil), reflecting differences in emulsion network strength associated with the fatty acid composition of the incorporated oils. Texture analysis revealed that linseed, thistle, and corn oil formulations were characterized by markedly lower consistency values (17.02–18.68 N·s) compared to all other samples (43.27–69.95 N·s), likely related to the high PUFA content of these oils and their effect on interfacial network organization, a finding consistent with their reduced T_2_ relaxation values indicating a more constrained water environment and denser interfacial network. LF-NMR droplet size analysis identified pomegranate oil as producing the finest emulsion microstructure (D = 2.653 µm), while avocado and olive oil formulations showed the highest proportions of large droplets, suggesting lower emulsification efficiency related to their high oleic acid content and elevated viscosity. From a nutritional perspective, linseed, walnut, and pomegranate oils showed favorable PUFA-to-SFA ratios and may be considered potential options for mayonnaise reformulation. Overall, the results demonstrate that the selection of functional oil type is a critical determinant of both the technological and nutritional quality of mayonnaise, and that LF-NMR provides a sensitive and non-destructive tool for characterizing emulsion microstructure in such complex food systems. The present study was primarily focused on compositional and physicochemical characterization, while aspects such as sensory acceptance, storage stability, and microbiological quality were beyond its scope. Nevertheless, the higher PUFA content of some oils suggests that their susceptibility to oxidation should be considered in future work.

## Figures and Tables

**Table 1:** Fatty acid composition (SFA, MUFA, PUFA) of functional oils used in mayonnaise enrichment (% *w*/*w*).

Oil	SFA (%)	MUFA (%)	PUFA (%)
Rapeseed	8	60	32
Sunflower	11	20	69
Grapeseed	10	16	74
Pomegranate	13	13	75
Almond	11	66	23
Cumin	19	29	52
Peanut	19	64	17
Soybean	16	24	60
Olive	14	73	11
Pumpkin	22	31	47
Walnut	16	8	75
Avocado	14	73	11
Hemp	12	20	68
Sesame	13	38	49
Linseed	9	18	73
Thistle	17	16	67
Corn	17	33	50

SFA—saturated fatty acids; MUFA—monounsaturated fatty acids; PUFA—polyunsaturated fatty acids.

## Data Availability

The original contributions presented in this study are included in the article/Supplementary Materials. Further inquiries can be directed to the corresponding author.

## Supplementary Materials

The following supporting information can be downloaded at https://www.mdpi.com/article/10.3390/foods15122184/s1: Table S1: Processing type and organic status of the vegetable oils used in mayonnaise formulation; Table S2: CIE Lab color parameters (L*, a*, b*) of functional oil-enriched mayonnaise formulations (D65 illuminant); Table S3: Textural parameters of functional oil-enriched mayonnaise formulations: Firmness (N), Consistency (N·s), Work of Cohesion (N·mm) and Cohesiveness (N·s); Table S4: Power Law parameters of functional oil-enriched mayonnaise formulations: Consistency index K (Pa·s^n^), Flow behavior index n (−), R^2^ apparent viscosity (η), R^2^ shear stress (τ); Table S5: LF-NMR droplet size distribution of functional oil-enriched mayonnaise: Mean radius (µm), volume fractions <2, 2–5, 5–10 µm (%).
