# Supplementary material for: Rheological and Physicochemical Properties of Mayonnaise Enriched with Functional Vegetable Oils: A Comparative Screening Study
Source: Foods. 2026 Jun 17;15(12):2184. doi: 10.3390/foods15122184 (PMC13297992; doi:10.3390/foods15122184)
Supplement: Supplementary file 1 [file foods-15-02184-s001.zip › foods-4317223-supplementary.pdf]

## Supplementary Material

# Rheological and Physicochemical Properties of Mayonnaise Enriched with Functional Vegetable Oils: A Comparative Screening Study

**Shakhnozakhon Gaipova [1,†], Umrbek Mavlanov [2,†] and Tomasz Pawel Czaja [2,*]**

[1] Department of Food and Perfumery-Cosmetic Products, Tashkent Institute of Chemical Technology, Navoi 32, Tashkent 100011, Uzbekistan
[2] Department of Food Science, University of Copenhagen, Rolighedsvej 26, 1958 Frederiksberg C, Denmark
* Correspondence: tomasz.czaja@food.ku.dk
† These authors contributed equally to this work.

**Table S1** Processing type and organic status of the vegetable oils used in mayonnaise formulation.

| Oil | Processing | Organic |
|---|---|---|
| Rapeseed | Refined | N.A |
| Sunflower | Refined | N.A |
| Grapeseed | Virgin | N.A |
| Pomegranate | Cold-pressed | Organic |
| Almond | Cold-pressed | Organic |
| Cumin | Cold-pressed | Organic |
| Peanut | Refined | N.A |
| Soybean | Refined | N.A |
| Olive | Extra Virgin | N.A |
| Pumpkin | Cold-pressed | Organic |
| Walnut | Cold-pressed | Organic |
| Avocado | Cold-pressed | Organic |
| Hemp | Cold-pressed | Organic |
| Sesame | N.A | N.A |
| Linseed | Cold-pressed | Organic |
| Thistle | Cold-pressed | Organic |
| Corn | Cold-pressed | Organic |

**Table S2** CIE Lab color parameters (L*, a*, b*) of functional oil-enriched mayonnaise formulations (D65 illuminant).

| Oil | L* | a* | b* | ΔE* |
|---|---|---|---|---|
| Control (Rapeseed) | 81.43 | -0.40 | 13.01 | - |
| Sunflower | 82.19 | -0.78 | 12.49 | 1.05 |
| Grapeseed | 81.36 | -1.03 | 13.33 | 1.35 |
| Pomegranate | 82.04 | -0.60 | 14.52 | 1.14 |
| Almond | 81.65 | -0.25 | 11.37 | 1.03 |
| Cumin | 79.59 | -0.77 | 20.49 | 7.77 |
| Peanut | 82.04 | -0.75 | 12.74 | 0.65 |
| Soybean | 81.12 | -1.10 | 12.61 | 0.59 |
| Olive | 81.44 | -0.44 | 16.61 | 3.64 |
| Pumpkin | 74.39 | -3.59 | 22.06 | 8.11 |
| Walnut | 82.31 | -0.37 | 13.38 | 1.34 |
| Avocado | 76.56 | -1.97 | 30.08 | 18.45 |
| Hemp | 75.38 | -1.86 | 25.55 | 14.50 |
| Sesame | 81.93 | -0.78 | 13.43 | 1.12 |
| Linseed | 82.31 | -0.77 | 13.45 | 1.02 |
| Thistle | 76.63 | -0.89 | 13.66 | 10.30 |
| Corn | 75.36 | -0.99 | 13.89 | 10.11 |

**Table S3** Textural parameters of functional oil-enriched mayonnaise formulations: Firmness (N), Consistency (N·s ), Work of Cohesion (N·mm) and Cohesiveness (N·s),

| Oil | Firmness | Consistency | Work of Cohesion | Cohesiveness |
|---|---|---|---|---|
| Control (Rapeseed) | 6.25 | 51.06 | 5.84 | 5.45 |
| Sunflower | 9.09 | 69.95 | 7.11 | 6.70 |
| Grapeseed | 8.27 | 60.48 | 6.99 | 7.07 |
| Pomegranate | 6.06 | 44.29 | 5.26 | 5.55 |
| Almond | 6.88 | 52.45 | 6.49 | 6.35 |
| Cumin | 5.89 | 43.27 | 5.31 | 4.79 |
| Peanut | 6.55 | 52.36 | 6.02 | 5.95 |
| Soybean | 6.64 | 49.97 | 5.84 | 6.00 |
| Olive | 6.50 | 51.88 | 6.20 | 6.21 |
| Pumpkin | 7.18 | 53.75 | 6.53 | 6.69 |
| Walnut | 6.02 | 46.11 | 5.39 | 5.60 |
| Avocado | 5.98 | 47.99 | 5.64 | 5.87 |
| Hemp | 6.34 | 49.59 | 5.89 | 5.91 |
| Sesame | 7.45 | 53.70 | 6.29 | 6.34 |
| Linseed | 6.77 | 17.02 | 5.74 | 4.68 |
| Thistle | 7.54 | 18.68 | 5.74 | 4.62 |
| Corn | 7.31 | 17.87 | 5.39 | 4.62 |

**Table S4** Power Law parameters of functional oil-enriched mayonnaise formulations: Consistency index K ($Pa \cdot s^n$), Flow behavior index n (−), $R^2$ apparent viscosity (η), $R^2$ shear stress (τ).

| Oil | K ($Pa \cdot s^n$) | n | $R^2$ η | $R^2$ τ |
|---|---|---|---|---|
| Control | 118.93 | 0.162 | 0.994 | 0.996 |
| Sunflower | 112.56 | 0.231 | 0.993 | 0.964 |
| Grapeseed | 130.63 | 0.194 | 0.992 | 0.951 |
| Pomegranate | 90.12 | 0.238 | 0.984 | 0.969 |
| Almond | 114.44 | 0.221 | 0.994 | 0.960 |
| Cumin | 92.10 | 0.240 | 0.985 | 0.966 |
| Peanut | 100.40 | 0.249 | 0.992 | 0.957 |
| Soybean | 114.79 | 0.213 | 0.994 | 0.966 |
| Olive | 104.31 | 0.232 | 0.993 | 0.968 |
| Pumpkin | 118.53 | 0.224 | 0.993 | 0.962 |
| Walnut | 92.05 | 0.229 | 0.997 | 0.988 |
| Avocado | 97.70 | 0.242 | 0.988 | 0.966 |
| Hemp | 109.70 | 0.234 | 0.993 | 0.979 |
| Sesame | 92.05 | 0.229 | 0.997 | 0.988 |
| Linseed | 104.84 | 0.195 | 0.995 | 0.992 |
| Thistle | 108.22 | 0.226 | 0.993 | 0.971 |
| Corn | 93.56 | 0.241 | 0.993 | 0.963 |

**Table S5** LF-NMR droplet size distribution of functional oil-enriched mayonnaise: Mean radius (μm), volume fractions < 2, 2–5, 5–10 μm (%).

| **Oil** | **Mean radius (D, μm)** | **< 2 μm (%)** | **2 to 5 μm (%)** | **5 to 10 μm (%)** |
|---|---|---|---|---|
| Control | 2.904 | 25.11 | 73.57 | 1.32 |
| Sunflower | 2.801 | 30.58 | 68.11 | 1.31 |
| Grapeseed | 2.810 | 31.75 | 66.46 | 1.79 |
| Pomegranate | 2.653 | 35.59 | 63.71 | 0.70 |
| Almond | 3.032 | 24.40 | 72.80 | 2.79 |
| Cumin | 2.735 | 30.46 | 68.90 | 0.64 |
| Peanut | 2.859 | 30.48 | 67.49 | 2.03 |
| Soybean | 2.892 | 26.55 | 71.95 | 1.50 |
| Olive | 3.200 | 23.19 | 71.63 | 5.15 |
| Pumpkin | 2.851 | 28.68 | 69.84 | 1.48 |
| Walnut | 2.858 | 28.60 | 69.85 | 1.55 |
| Avocado | 3.182 | 27.38 | 66.13 | 6.40 |
| Hemp | 2.809 | 31.36 | 67.06 | 1.58 |
| Sesame | 2.937 | 30.76 | 65.91 | 3.32 |
| Linseed | 3.203 | 19.72 | 76.34 | 3.94 |
| Thistle | 2.839 | 25.37 | 73.88 | 0.75 |
| Corn | 3.014 | 22.47 | 75.52 | 2.01 |
